# Patient Knowledge and Attitudes towards Genetic Testing in Parkinson's Disease Subjects with Deep Brain Stimulation

**DOI:** 10.1155/2019/3494609

**Published:** 2019-04-21

**Authors:** Avram Fraint, Bichun Ouyang, Leonard Verhagen Metman, Carolyn Jones, Deborah A. Hall, Karen Marder, Gian Pal

**Affiliations:** ^1^Department of Neurological Sciences, Rush University Medical Center, Chicago, IL, USA; ^2^Department of Neurology and the Taub Institute, Columbia University Medical Center, New York, NY, USA

## Abstract

**Objectives:**

As genetic testing is becoming more widely commercially available for Parkinson's disease (PD) and may have implications regarding clinical outcomes for deep brain stimulation (DBS) and other therapies, we aimed to determine patient knowledge and attitudes towards genetic testing.

**Methods:**

A sample of 88 PD subjects with bilateral STN-DBS completed a Genetic Attitudes Questionnaire (GAQ). Knowledge and attitudes towards genetic testing were assessed.

**Results:**

The mean percent of correct responses regarding genetic testing knowledge was 58.5%. Nearly 90% of subjects were unfamiliar with Genetic Information Nondiscrimination Act (GINA). The most important reasons subjects cited in deciding whether to undergo genetic testing included (1) to be a candidate for clinical trials if positive, (2) to learn that they do not carry a mutation, and (3) because a healthcare provider had recommended it. Individuals who influence decision-making include spouses and children. About 88% of subjects would share results with spouses, children, and siblings.

**Discussion:**

These results reveal that there is a major knowledge gap regarding genetic testing in PD and the implications of testing results on treatment, work, insurance, and privacy. Also, subjects would mainly seek genetic testing to participate in clinical trials, with spouses and children being the key stakeholders in decision-making.

## 1. Introduction

About 9,000 patients with Parkinson's disease (PD) undergo deep brain stimulation (DBS) implantation annually worldwide [1]. Approximately 26–29% of PD patients who undergo DBS have a mutation in one of three genes: *glucocerebrosidase (GBA), leucine-rich repeat kinase 2 (LRRK2)*, and *parkin (PRKN)* [2, 3]. In general, the majority of PD cases are sporadic (90%), and monogenic forms of the disease account for approximately 3–5% of sporadic PD cases [4]. *GBA*, a risk factor gene for PD, is found in approximately 7% of the general PD population [5]. Series of PD patients who undergo DBS are thought to be enriched with genetic forms of the disease since those who opt for DBS tend to have a younger age of onset compared to the general population, clear levodopa responsiveness, and complications associated with dopaminergic treatment, namely, motor fluctuations and dyskinesia [3].

To our knowledge, genetic testing is not part of routine clinical care at the present time for PD as it does not influence clinical decision-making. This is true of our center and other specialty centers throughout the world [6]. However, genetic testing for PD is becoming more widely available commercially worldwide through direct-to-consumer (DTC) testing, namely, 23andMe [7]. Genetic counseling in instances of DTC testing is recommended but optional, and it is not clear what knowledge patients are receiving and retaining in these cases. Furthermore, clinicians are able to offer genetic testing through standardized commercial panels in the US and worldwide, but this is not part of routine care [6].

As genotype-phenotype correlations become more clear, it is possible that genetic mutation status may soon play a role in clinical decision-making for treatments and interventions such as DBS [8]. For instance, it has recently been reported that *LRRK2* G2019S mutation carriers have greater improvement from DBS compared with nonmutation carriers [9]. Also, Lythe et al. [10] have reported that *GBA* mutation carriers with DBS have more significant cognitive impairment and reduced quality of life compared with nonmutation carriers with DBS. In fact, the effects of STN-DBS in *GBA* mutation carriers are currently being investigated in a prospective longitudinal clinical trial assessing cognitive, motor, and quality of life outcomes (Clinicaltrials.gov NCT03234478). There are also ongoing clinical trials aimed at using pharmacologic agents to alter the progression of GBA-associated PD, and similar trials will be starting for *LRRK2*-associated PD [11]. This issue of linking genetic testing with clinical outcomes is relevant worldwide, but availability of such genetic testing may vary based on the specific health system for each individual country.

With these observations and the growing interest in gene-DBS interactions and gene-based treatments, it is critical to understand PD patients' knowledge base regarding genetic testing and their expectations regarding how this genetic information may or may not impact their treatment. We assessed knowledge and attitudes towards genetic testing in a consecutive sample of PD subjects who had already undergone STN-DBS.

## 2. Methods

Approval for the study was obtained from the Rush University Medical Center Institutional Review Board, and all patients signed informed consent for study participation. Consecutive PD patients with bilateral STN-DBS were recruited from the Movement Disorders clinic of Rush University Medical Center in Chicago, Illinois. The main inclusion criteria were as follows: (1) clinical diagnosis of idiopathic PD, (2) implantation with bilateral STN-DBS, (3) agreement to attempt completing a Genetic Attitudes Questionnaire (GAQ), and (4) English speaking to ensure comprehension of the GAQ. Patients were not given any specific information regarding genetic testing either before or after their surgery as the goal was to obtain their baseline level of knowledge and attitudes towards genetic testing. Furthermore, genetic testing is not part of routine clinical care of PD [6] at our center at the present time, and therefore, genetic counseling is not routinely offered.

Demographic data including current age, sex, age at onset of PD symptoms, disease duration, family history of PD, and ethnicity were collected. The GAQ has been previously described [12, 13] and was administered in person or via telephone. We focused on two sections of the GAQ (Supplementary Materials): (A) knowledge base related to genetic testing (13 questions) and (B) attitudes towards genetic testing (24 questions). “True/false” and “yes/no” question formats were employed to assess knowledge related to genetic testing. To assess attitudes towards genetic testing, 4-item forced response scales and “yes/no” questions were employed. For instance, when subjects were asked how important specific reasons were in their decision regarding genetic testing, response options included “not important at all,” “somewhat important,” “very important,” or “not applicable.” The questions that comprise the GAQ have been previously published [14]. For all subjects, we quantified (1) correct responses to knowledge questions and (2) responses regarding attitudes towards genetic testing.

## 3. Results

### 3.1. Demographics

One hundred subjects were enrolled in the study, and GAQ data were available for eighty-eight subjects (88%). The remaining 12 who were recruited but not included in the final analysis were lost to follow-up for completion of the GAQ despite multiple attempts to make contact via telephone. Demographic characteristics for the remaining 88 subjects with complete data are summarized in Table 1.

### 3.2. Knowledge regarding Genetic Testing

**Figure 1:**
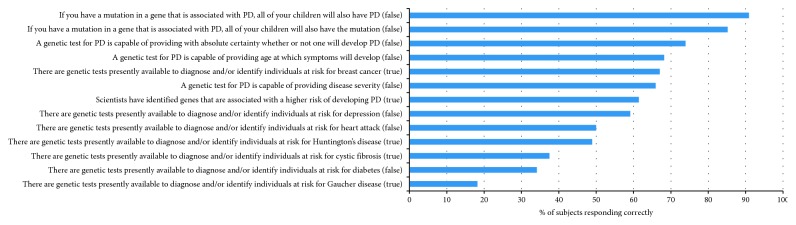
Knowledge regarding genetic testing. Percentage of correct responses to genetic knowledge questions in all subjects (*n* = 88).

For the entire group, the mean percent of correct responses regarding genetic testing knowledge was 58.5%. Approximately 64% of subjects were aware that genes have been identified that confer a higher risk of developing PD (Figure 1). The majority of subjects correctly indicated that there is no genetic test currently available that accurately indicates that a person will or will not develop PD (73.9% correctly responded), that determines the age of PD symptom onset (68.2% correctly responded), or determines disease severity (65.9% correctly responded). A majority of the cohort correctly answered that identifying probands with a mutation does not ensure that their children would have the same mutation (85.2%) or that their child would definitely develop PD (90.9%). Only 49% of respondents correctly indicated that there is a genetic test for Huntington's disease, 37% were aware of testing for cystic fibrosis, and only 18% of subjects were aware of genetic testing for Gaucher disease (GD), a known risk factor for PD. Nearly 90% of subjects were not familiar with the Genetic Information Nondiscrimination Act (GINA), and about 36% of subjects were unfamiliar with Health Insurance Portability and Accountability Act (HIPAA) despite having signed a HIPAA consent at the time of enrollment.

### 3.3. Attitudes towards Genetic Testing

**Figure 2:**
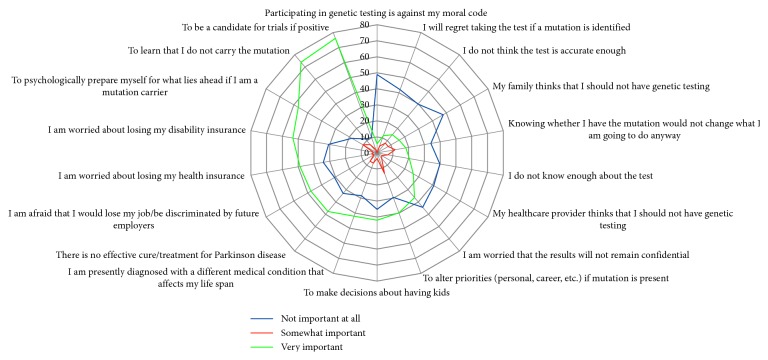
Attitudes towards genetic testing.

Over 50% of subjects listed the following factors as very important factors regarding their desire for genetic testing: “to be a candidate for clinical trials if positive,” “to learn that I do not carry the mutation,” “my healthcare provider thought I should have genetic testing,” “to psychologically prepare myself for lies ahead if I am a mutation carrier,” and “I am worried about losing my disability insurance” (Figure 2). When subjects were asked “If a genetic test existed to determine how likely you were to benefit from a particular medication to lessen/improve Parkinson disease symptoms,” 58% responded that they would definitely take the test, and 33% responded they would only take the test if covered by insurance. When subjects were asked “If a genetic test existed to determine how likely you were to develop side effects from a particular medication to treat your Parkinson disease,” 52% responded that they would definitely take the test, and 37% responded they would only take the test if covered by insurance.

**Figure 3:**
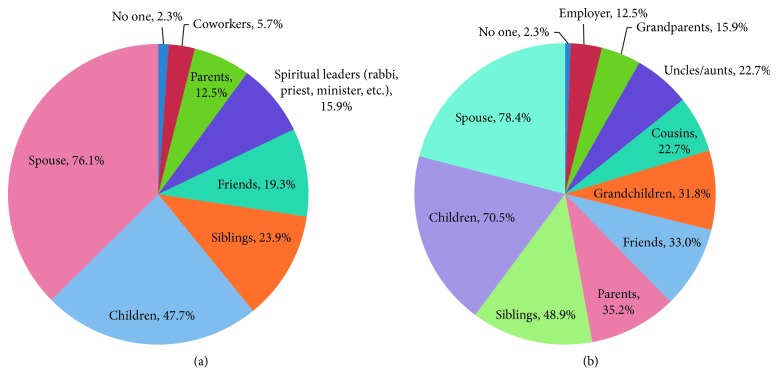
(a) Individuals who influence patient's decision-making. (b) Individuals with whom subjects would share results.

Over 50% of subjects noted that the following individuals were influential in their decision-making: spouse (76%) and children (47%) (Figure 3(a)). Over 88% of subjects would share results of their testing regardless of the result, while 60% of subjects responded they would only share the result if they were mutation negative. Over 50% of subjects responded they would share results with their spouse (78%) and children (70%) (Figure 3(b)).

## 4. Discussion

Genetic testing provides the opportunity to correlate individual genotype with clinical outcome of therapies such as DBS [8]. Before this can be successfully implemented, it is important to understand patient knowledge and attitudes regarding genetic testing. This is the first study to examine these factors in a PD population who have already undergone DBS. We specifically chose this population since they have firsthand experience regarding the benefits, limitations, and heterogeneity of outcomes associated with the DBS therapy. With this personal experience regarding DBS, these subjects offer a unique perspective on reasons for obtaining genetic testing. Compared with the subjects who completed the GAQ, as reported by Gupte et al. [13], our cohort was comparable in age, but subjects in our cohort had a longer disease duration (16.5 vs. 9.8 years, respectively) and earlier age of onset (age 47.0 vs. 58.8 years, respectively) at the time of GAQ completion.

### 4.1. Knowledge regarding Genetic Testing

The mean percent of correct responses regarding genetic testing knowledge was 58.5%, which is comparable to percent of correct genetic testing knowledge questions regarding breast cancer amongst Caucasians [15]. Approximately 86% of our sample were Caucasian, and knowledge regarding genetic testing has been reported to be lower in other ethnic groups such as Hispanics and African Americans [16–19]. Also, our population was highly educated, with over 90% of subjects with a high school degree or greater level of education, and over 50% of subjects having a college or postcollege degree. Given the increasing commercial availability of direct-to-consumer genetic testing for PD through services such as “*23andme*” [20] that do not provide mandatory formal genetic counseling along with testing results, our results identify a critical need to increase knowledge regarding genetic testing among PD patients and their families. Furthermore, targeted PD genetic testing knowledge among minority groups remains to be further studied. Only 10% of subjects were familiar with GINA, and 53% of subjects were concerned about losing their disability insurance, indicating that there is a major knowledge gap regarding the implications of genetic testing results regarding work, insurance, and privacy. Lastly, only 18% of subjects were familiar with genetic testing for Gaucher's disease, which is caused by homozygous mutations in the *GBA* gene. Mutation in *GBA* is the most common genetic risk factor for PD. Explaining that heterozygous mutations predispose an individual to an increased risk of PD and homozygous mutations cause yet another disease, Gaucher's disease [21], is a large amount of information for patients and families to comprehend and digest. Knowledge of *GBA* and the implications of testing for this gene will be particularly important for patients and families to understand as genetic testing becomes more widely available and more of these mutation carriers are identified.

We acknowledge that the data provided here are from a single medical center. However, we posit that the knowledge gap seen in our patients may be reflective of the general PD population. This is likely given that there are currently no standardized set of tools that are used systematically by clinicians to educate patients regarding genetic testing in PD. Patients typically rely on web-based resources, patients' associations, and their clinicians to provide them with information regarding PD genetics, but these resources certainly vary in their availability, quality, and comprehensiveness. Therefore, PD patients and their families would likely benefit from development of educational tools and resources regarding genetic testing.

### 4.2. Attitudes towards Genetic Testing

The most important reason subjects cited in deciding whether to undergo genetic testing included being a candidate for trials if they carry a mutation. Gupte et al. [13] also reported that eligibility for clinical trials was one of the top reasons subjects would pursue genetic testing. With the advent of precision-based therapies for cancer and autoimmune disease, patients are increasingly seeking out personalized medicine treatments and are willing to consider experimental therapies [22]. Subjects also responded that learning that they “do not carry the mutation” was an important consideration regarding genetic testing. Oncology studies that examine psychosocial and behavioral outcomes of genetic testing demonstrate limited adverse psychological outcomes associated with testing [23]. However, the psychosocial impact of receiving genetic testing results in PD is unknown since widespread genetic testing is not clinically employed as of yet.

Approximately 52% of subjects would opt for genetic testing if it could predict side effects of a treatment and 58% of subjects would opt for genetic testing if it could predict treatment benefit. We had anticipated that the vast majority of subjects would have wanted genetic testing if it could help maximize their outcomes. Limitations for this study include the fact that all subjects in our study already had longstanding DBS implantation which may bias our sample as these subjects may have been satisfied with their current level of functionality, so they may believe that genetic testing would not add more to their care. Indeed, satisfaction with DBS is high in patients with greater than 5 years of therapy [24] though there are no data currently available regarding satisfaction based on the genetic status. Furthermore, we did not assess for apathy in this vulnerable population [25], which may also have an impact on desire for genetic data. Lastly, the study is limited by our assessment of only DBS subjects and future studies should be designed to compare subjects with and without DBS. However, the utility of genetic testing in DBS is a rapidly evolving issue, and this study is the first of its kind to assess knowledge and attitudes towards genetic testing in the context of potential clinical application in PD.

We found that individuals who influence decision-making include spouses and children, and about 88% of subjects would share results with relatives, particularly with spouses, children, and siblings. In our study, we focused on knowledge and attitudes towards genetic testing in patients with PD, but it is also important to engage spouses, children, family members, and other key stakeholders in decision-making regarding genetic testing.

### 4.3. Future Directions

PD patients and their families will likely benefit from development of educational tools and resources regarding genetic testing, given the clear knowledge gap identified in this study as results may have significant implications for work, insurance, and privacy. This will be particularly important if genetic testing becomes incorporated into the treatment algorithm for therapies such as DBS. Also, it is important for clinicians to engage the key stakeholders in decisions regarding genetic testing and the implications of such testing. There are efforts to make genetic testing more easily accessible to patients through organizations, such as the Michael J. Fox Foundation, since patients may qualify for targeted treatments in the form of clinical trials. It will be particularly important to counsel patients regarding the implications and meaning of genetic testing results in such cases as such gene-based trials become increasingly common in PD.

Lastly, the issue of genotype-phenotype correlations regarding DBS outcomes extends to other movement disorders as well, particularly dystonia. For instance, studies have suggested that individuals with *TOR1A* mutations typically respond better to pallidal DBS than individuals with *THAP1* mutations or undetermined genetic causes [26]. As genotype becomes increasingly linked to clinical outcomes for a variety of diseases and therapies such as DBS, it will be critically important to educate patients, families, and clinicians alike, regarding the implications and limitations of genetic testing.

## 5. Conclusions

As the field of Parkinson's disease and movement disorder genetics moves towards a precision-medicine approach to treatment of the disease, patients and key stakeholders require education regarding the implications of genetic testing in order to make informed healthcare decisions.

## Figures and Tables

**Table 1:** Demographics and subject characteristics.

Age, mean (SD)	63.67 (7.72)
Sex, *n* (%)	
** **Male	59 (67.0)
** **female	29 (33.0)
Ethnicity, *n* (%)	
** **Caucasian	76 (86.4)
** **AA	1 (1.1)
** **Hispanic	2 (2.3)
** **Ashkenazi Jewish	1 (1.1)
** **Others	3 (3.4)
** **Missing	6 (5.7)
Education, *n* (%)	
** **Postcollege degree	23 (26.1)
** **College degree	24 (27.3)
** **Some college	16 (18.2)
** **High school diploma	20 (22.7)
** **K-8	1 (1.1)
** **Not available	4 (4.5)
Age of onset, mean (SD)	47.02 (9.24)
Disease duration, mean (SD)	16.54 (6.28)
UPDRS-III, mean (SD)	24.24 (11.11)
1st degree relative with PD, *n* (%)	
** **No	66 (75.0)
** **Yes	22 (25.0)

## Data Availability

The data used to support the findings of this study are available from the corresponding author upon request.

## Supplementary Materials

Supplementary Materials The GAQ items that were assessed are shown in the supplementary material.
