# Supplementary material for: Patient Knowledge and Attitudes towards Genetic Testing in Parkinson's Disease Subjects with Deep Brain Stimulation
Source: Parkinsons Dis. 2019 Apr 21;2019:3494609. doi: 10.1155/2019/3494609 (PMC6501170; doi:10.1155/2019/3494609)
Supplement: Supplementary Materials — The GAQ items that were assessed are shown in the supplementary material. [file 3494609.f1.pdf]

Supplementary Material. Genetics Attitudes Questionnaire (GAQ)

Knowledge regarding genetic testing:

Please indicate whether you believe the statements below to be TRUE or FALSE.

1. Scientists have identified genes that are associated with a higher risk of developing Parkinson disease.
2. A genetic test for Parkinson disease is capable of providing the age at which symptoms will develop.
3. A genetic test for Parkinson disease is capable of predicting disease severity.
4. A genetic test for Parkinson disease is capable of predicting with absolute certainty whether or not one will develop Parkinson disease
5. If you have a mutation in a gene that is associated with Parkinson disease, all of your children will also have the mutation.
6. If you have a mutation in a gene that is associated with Parkinson disease, all of your children will also have Parkinson Disease.
7. There are genetic tests presently available to diagnose and/or identify individuals at risk for depression.
8. There are genetic tests presently available to diagnose and/or identify individuals at risk for cystic fibrosis.
9. There are genetic tests presently available to diagnose and/or identify individuals at risk for breast cancer.
10. There are genetic tests presently available to diagnose and/or identify individuals at risk for heart attack.
11. There are genetic tests presently available to diagnose and/or identify individuals at risk for diabetes.
12. There are genetic tests presently available to diagnose and/or identify individuals at risk for Huntington's disease.
13. There are genetic tests presently available to diagnose and/or identify individuals at risk for Gaucher disease.

Attitudes towards genetic testing:

	Not important at all	Somewhat important	Very important	Not applicable
To learn that I do not carry the mutation.				
To make decisions about having (more) children.				
To alter priorities (personal, career, etc) if mutation is present.				
To be able to psychologically prepare myself for what lies ahead if the test reveals that I carry the mutation.				
If the test reveals that I carry the mutation, I would want to be identified early on so that I could be eligible for Parkinson prevention/treatment trials present and future.				
I will regret taking the test if a mutation is identified.				
Participating in genetic testing is against my personal moral code.				
Knowing whether I have the mutation would not change what I am going to do anyway.				
There is no effective cure/treatment for Parkinson disease.				
I do not know enough about the test.				
I do not think the test is accurate enough.				
My family thinks that I should not have genetic testing.				
My healthcare provider thinks that I should not have genetic testing.				
I am presently diagnosed with a different medical				

condition that affects my life span.				
I am worried that the results will not remain confidential.				
I am worried about losing my health insurance.				
I am worried about losing my disability insurance.				
I am afraid that I would lose my job/be discriminated by future employers.				

	Definitely take the test	Definitely not take the test	Take the test only if covered by insurance	Unsure
If a genetic test existed to determine how likely you were to benefit from a particular medication to lessen/improve Parkinson disease symptoms, you would				
If a genetic test existed to determine how likely you were to develop side effects from a particular medication to treat your Parkinson disease				

What people in your life influence your decision making?

	Yes	No
No one		
Coworkers		
Parents		
Spiritual leader (rabbi, priest, minister, etc.)		
Friends		
Siblings		
Children		
Spouse		

	Yes	No
If you had genetic testing, would you share the results of your test with relatives regardless of the result?		
If you had genetic testing would you share the results of your test with relatives only if the test indicated that you did not have a mutation?		

If you would share results, whom would you share them with?

	Yes	No
No one		
Employer		
Grandparents		
Aunts/Uncles		
Cousins		
Grandchildren		
Friends		

Parents		
Siblings		
Children		
Spouse		
